# Investigating whether H5N1 is a risk to human populations in Brazil

**DOI:** 10.1590/0037-8682-0056-2024

**Published:** 2024-07-29

**Authors:** Claudio Bruno Silva de Oliveira, Joelma Maria de Araújo Andrade, Shahina Akter, Maria Karolaynne da Silva, Umberto Laino Fulco, Jonas Ivan Nobre Oliveira

**Affiliations:** 1 Hospital Pediátrico Maria Alice Fernandes, Natal, RN, Brasil.; 2 Hemocentro Dalton Cunha, Natal, RN, Brasil.; 3 Bangladesh Council of Scientific & Industrial Research (BCSIR), Dhaka, Bangladesh.; 4 Universidade Federal do Rio Grande do Norte, Departamento de Biofísica e Farmacologia, Natal, RN, Brasil.

**Dear Editor,**

The increasing incidence of the avian influenza subtype H5N1 virus infection, initially observed in Europe and now more frequently in Latin America, has raised significant concerns. This outbreak has primarily affected wildlife along the coast of several regions, with some reports of human transmission. This type of infection rarely occurs in Brazil, but may be underreported, indicating a possible increase in prevalence. This letter raises critical concerns and proposes strategies to mitigate the associated public health risks.

The occurrence of H5N1 virus infection has already been identified, with some cases observed since 1996^1^. This situation has escalated globally since 2021, marked by the widespread prevalence of the highly pathogenic avian influenza A (HPAI) 2.3.4.4b lineage, which has affected several populations of wild and domestic birds due to its high mortality rate^1^ ^,^ ^2^.

Although there is great concern about cases in birds due to the numerous deaths caused by ecological and commercial losses^2^ ^,^ ^3^, we mainly focused on the risks to public health. Although originally found in birds, adaptation of this virus to mammals has been demonstrated in some European countries. Natural selection will always be a relevant factor in this context, as it facilitates adaptive mutations in polymerase proteins, enabling them to function in different hosts, such as mammals, as observed in Italy^4^ and the Netherlands, where the authors observed that mutations with evolutionary gain occurred in the foxes investigated^5^.

The H5N1 virus infection causes various symptoms in humans, ranging from asymptomatic or mild respiratory infections to severe infections. The condition can rapidly progress to pneumonia, acute respiratory distress syndrome, shock, and even death. Human infections are primarily associated with direct or indirect contact with infected birds, live or dead birds, or contaminated environments. Since 2003, epidemiological data have recorded 873 individuals who contracted H5N1 virus infections, resulting in 458 deaths^6^.

Domańska-Blicharz et al.^7^ observed that infected cats in Poland experienced severe consequences of the disease, including neurological and respiratory damage and death. Large quantities of the viral genome were detected in various specimens analyzed in these cats. The same damage and viral distribution patterns were found in ferrets, demonstrating that these are characteristic features of the 2.3.4.4b lineage^7^ ^,^ ^8^.

Most studies have observed mutations in PB2 (E627K and K526R) proteins, which serve as molecular markers for mammalian adaptation to the virus^9^. These mutations have demonstrated significant activity in *in vitro* and animal studies.

Researchers have expressed concern regarding the efficiency of clearing the infection, particularly focusing on the direct source of infection. Studies on cats have hypothesized that contaminated poultry meat may have inadvertently entered the food chain, as very similar genotypes were observed in large areas. This finding is especially troubling as it suggests that infection from wild animals may not be the greatest risk for spread^7^.

A consistent increase in the number of individuals with H5N1 virus infection has been observed in the Americas, initially in North America^10^, and subsequently in Peru, Chile, Argentina, Uruguay, and Brazil^3^ ^,^ ^6^ ^,^ ^11^. The disease was first detected in seabirds and later spread to various mammals. Leguia et al.^3^ have warned that HPAI poses a major threat to the extinction of local species in Peru.

The World Health Organization (WHO)^6^ has raised concerns about a confirmed H5N1 virus infection in an individual from Chile, identified as the clade 2.3.4.4b virus. However, analysis of the contact cases yielded negative results. Although this was an isolated case, the potential of this strain to spread among humans remains a cause of concern. Preliminary results of the epidemiological investigation of this patient suggest that the most plausible transmission route was environmental exposure, as a large number of dead marine mammals and wild birds were found near the patient’s residence^6^.

In addition to the aforementioned mutations, other variants should be monitored to closely track the evolution of the virus. The most important mutations that require attention are PA M861I and NS1 D26K, identified in samples from Peru and Chile^3^ ^,^ ^6^. Furthermore, mutations such as PB2-E627K, PB2-K702R, PB2-T271A, and PB2-D701N, which are associated with mammalian host adaptation and increased transmission, require continuous scrutiny^9^. Epidemiological surveillance bodies across South American countries, including Brazil, must systematically monitor these mutations due to their evolutionary advantages in mammalian hosts, affecting both replicative capacity and pathogenicity.

The observed potential for HPAI A/H5N1 transmission between mammals significantly increases concerns regarding the spread of the disease to humans, as previously documented^6^, followed by massive transmission among humans. Previous cases of infections in humans have resulted in death, prompting the WHO to declare that the current zoonotic threat of HPAI A/H5N1 remains high.

Considering that the primary source of new infections appears to be migratory birds^11^, Somenzari et al.^12^ reported that many migratory birds move between South American countries where the virus is established, such as Peru, Argentina, Chile, and Brazil. Thus, some of these birds could continuously introduce the virus into Brazil, underscoring the importance of monitoring mortality in these bird populations. Reischak et al.^11^ found a 99% similarity between strains from Chile and Peru in Cabot’s terns in Brazil, highlighting concerns regarding the migration of infected birds and the ongoing introduction of new viral strains.

Although the H5N1 virus infection occurring in Brazil has predominantly affected seabirds, more effective surveillance is needed to prevent the entry and spread of this virus in mammals. Rapid identification by competent bodies and the prompt implementation of necessary protective measures are essential.

Therefore, if H5N1 virus infection occurs among humans, the following measures should be employed: 1. immediate disposal of animals that have come into contact with infected birds to prevent further transmission, 2. provision of specific and immediate training for employees responsible for urban cleaning and those involved in the disposal of confirmed infected carcasses, 3. provision of necessary safety equipment to workers who will be directly involved in the disposal process to protect them from potential exposure, and 4. implementation of molecular diagnostic tools to detect H5N1 clades 2.3.4.4b, as this lineage poses the greatest potential risk.
